# Supplementary material for: Primary SARS-CoV-2 exposure by vaccination or infection shapes immune responses to omicron variants among a Spanish cohort
Source: Nat Commun. 2025 Dec 13;17:863. doi: 10.1038/s41467-025-67577-9 (PMC12827448; doi:10.1038/s41467-025-67577-9)
Supplement: Supplementary file 1 — Supplementary Information [file 41467_2025_67577_MOESM1_ESM.pdf]

Supplementary material

Table of Contents

Supplementary eTable 1. Additional information on Infection history before T10 and T11 timepoints 2

Supplementary eFigure 1. Correlations for IgA and IgG to the different RBDs (receptor binding domain) 3

Supplementary eFigure 2. Simple linear fit between number of exposures and antibody levels (Log_{10}MFI), stratified by the first exposure (infection/vaccine). MFI: Median fluorescence intensity. 5

Supplementary eTable 2. Mean difference on antibodies levels at timepoints T10 and T11 in the first-vaccinated vs the first-infected for infection and vaccine covariates from the main model (Model 5). 7

Supplementary eFigure 3. Percent change on IgA and IgG anti-RBD/anti-S ratio at timepoints T10 and T11 in first-vaccinated group minus first-infected group*. RBD: Receptor binding domain. S: Spike. 9

Supplementary eFigure 4. Comparison of different methods of p-value correction for multiplicity in the analysis of full sequence history of previous infections and vaccines. 10

Supplementary eFigure 5. Longitudinal Wuhan antibody levels against nucleocapsid (N), receptor-binding domain (RBD), and spike (S) proteins overtime applying different parametrizations and models (sensitivity analyses) 11

Supplementary eFigure 6. Magnitude of T-cell responses at T11 to S and N+M Wuhan antigens by first exposure groups. T: Timepoint. 14

Supplementary eTable3. Unadjusted and adjusted Cox Proportional Hazard model for the association between first exposure (first-vaccinated/first-infected) and breakthrough infections 15

Supplementary eFigure 7. Correlation between anti-RBD and neutralizing antibodies measured in a subset of individuals (n=89) from the cohort at T11. RBD: receptor binding domain. T: timepoint. 16

Supplementary eTable 4. Summary of linear mixed models used to estimate the association between first exposure (i.e., First-vaccinated *minus* First-infected) and anti-RBDs. RBD: Receptor binding domain. 17

Supplementary eFigure 8. Variance Inflation Factors (VIFs) from the main model (M5) for IgA and IgG and the seven RBDs. RBD: Receptor binding domain. 18

Supplementary eTable 5. Parametrizations for the antibody kinetics for IgA and IgG against Wuhan with anti-S, anti-N and anti-RBD antibodies using the data available from T5 to T11. S: Spike. N: Nucleocapsid. RBD: Receptor binding domain. T: Timepoint. 19

Supplementary eTable 1. Additional information on Infection history before T10 and T11 timepoints

	T10			T11		
	**First-infected (n=197)**	**First-vaccinated (n=160)**	**p-value**	**First-infected (n=197)**	**First-vaccinated (n=160)**	**p-value**
**Infections**						
Number of previous asymptomatic infections, mean (SD)	0.41 (0.56)	0.42 (0.55)	0.831	0.41 (0.56)	0.42 (0.55)	0.831
Number of previous asymptomatic infections, n (%)			0.903			0.903
0	124 (62.9)	98 (61.3)		124 (62.9)	98 (61.3)	
1	66 (33.5)	57 (35.6)		66 (33.5)	57 (35.6)	
2	7 (3.6)	5 (3.1)		7 (3.6)	5 (3.1)	
Number of previous asymptomatic non-Omicron infections, n (%)			0.384			0.384
0	163 (82.7)	137 (85.6)		163 (82.7)	137 (85.6)	
1	32 (16.2)	23 (14.4)		32 (16.2)	23 (14.4)	
2	2 (1.0)	0 (0.0)		2 (1.0)	0 (0.0)	
Number of previous asymptomatic Omicron infections, n (%)			0.541			0.541
0	154 (78.2)	117 (73.1)		154 (78.2)	117 (73.1)	
1	42 (21.3)	42 (26.2)		42 (21.3)	42 (26.2)	
2	1 (0.5)	1 (0.6)		1 (0.5)	1 (0.6)	
Previous BA.1 infections,* n (%) (one infection)	37 (18.8)	49 (30.6)	0.013	37 (18.8)	49 (30.6)	0.013
Previous BA.2 infections,* n (%)(one infection)	44 (22.3)	41 (25.6)	0.548	44 (22.3)	41 (25.6)	0.548
Previous BA.4.5 infections,* n (%)(one infection)	27 (13.7)	41 (25.6)	0.007	27 (13.7)	41 (25.6)	0.007
Number of previous BQ.1 infections,* n (%)			0.371			0.589
0	161 (81.7)	121 (75.6)		151 (76.6)	115 (71.9)	
1	35 (17.8)	38 (23.8)		45 (22.8)	44 (27.5)	
2	1 (0.5)	1 (0.6)		1 (0.5)	1 (0.6)	
Previous XBB infections, n (%)(one infection)	0 (0.0)	1 (0.6)	0.917	7 (3.6)	3 (1.9)	0.527

* includes symptomatic and asymptomatic infections. The p-values were two-sided. The tests applied where Chi-square/Fisher for categories and Mann-Whitney for continuous variables.

Supplementary eFigure 1. Correlations for IgA and IgG to the different RBDs (receptor binding domain)

A - Between IgA, IgG and IgA x IgG at T10 and T11

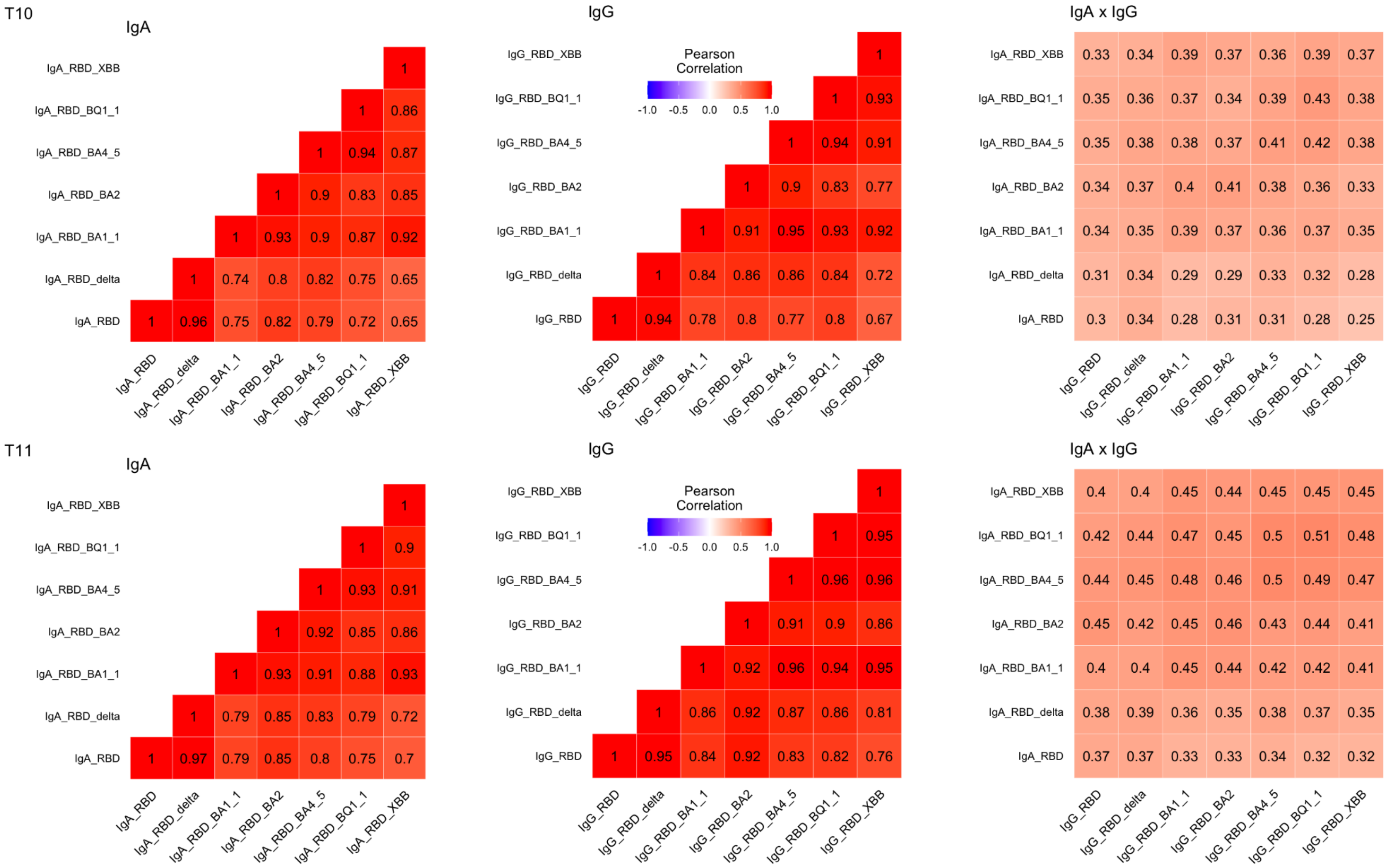

B - Between T10 and T11 for IgA and IgG

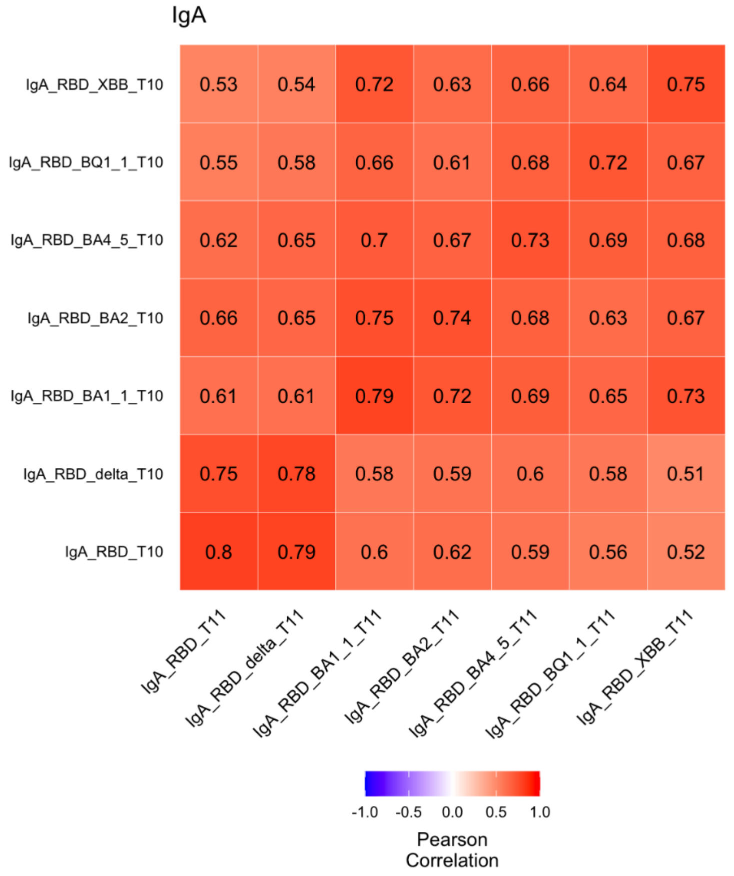

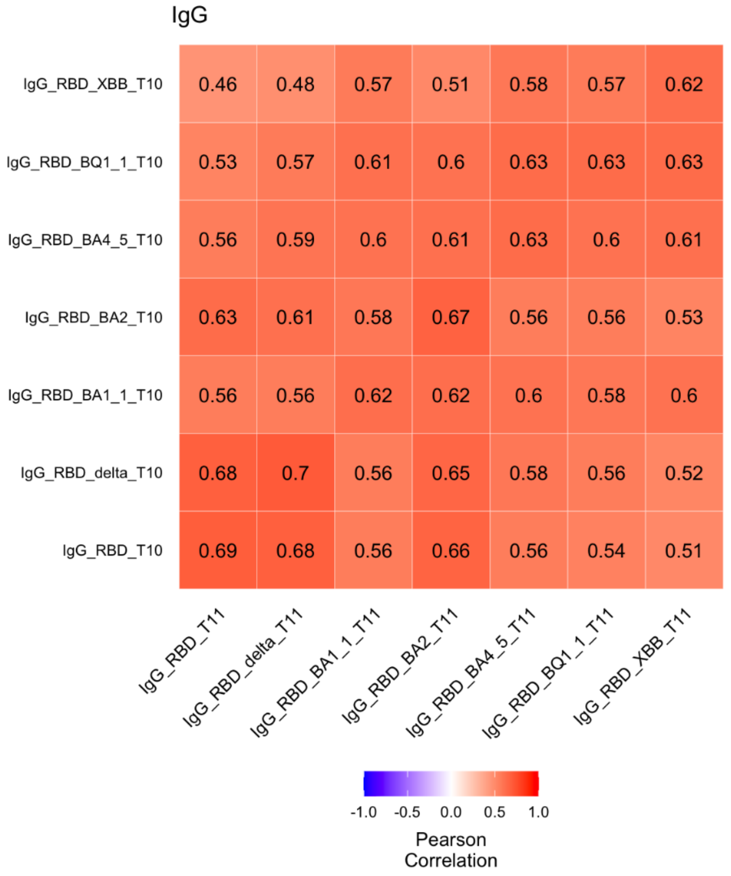

Supplementary eFigure 2. Simple linear fit between number of exposures and antibody levels (Log_{10}MFI), stratified by the first exposure (infection/vaccine). MFI: Median fluorescence intensity.

At T10

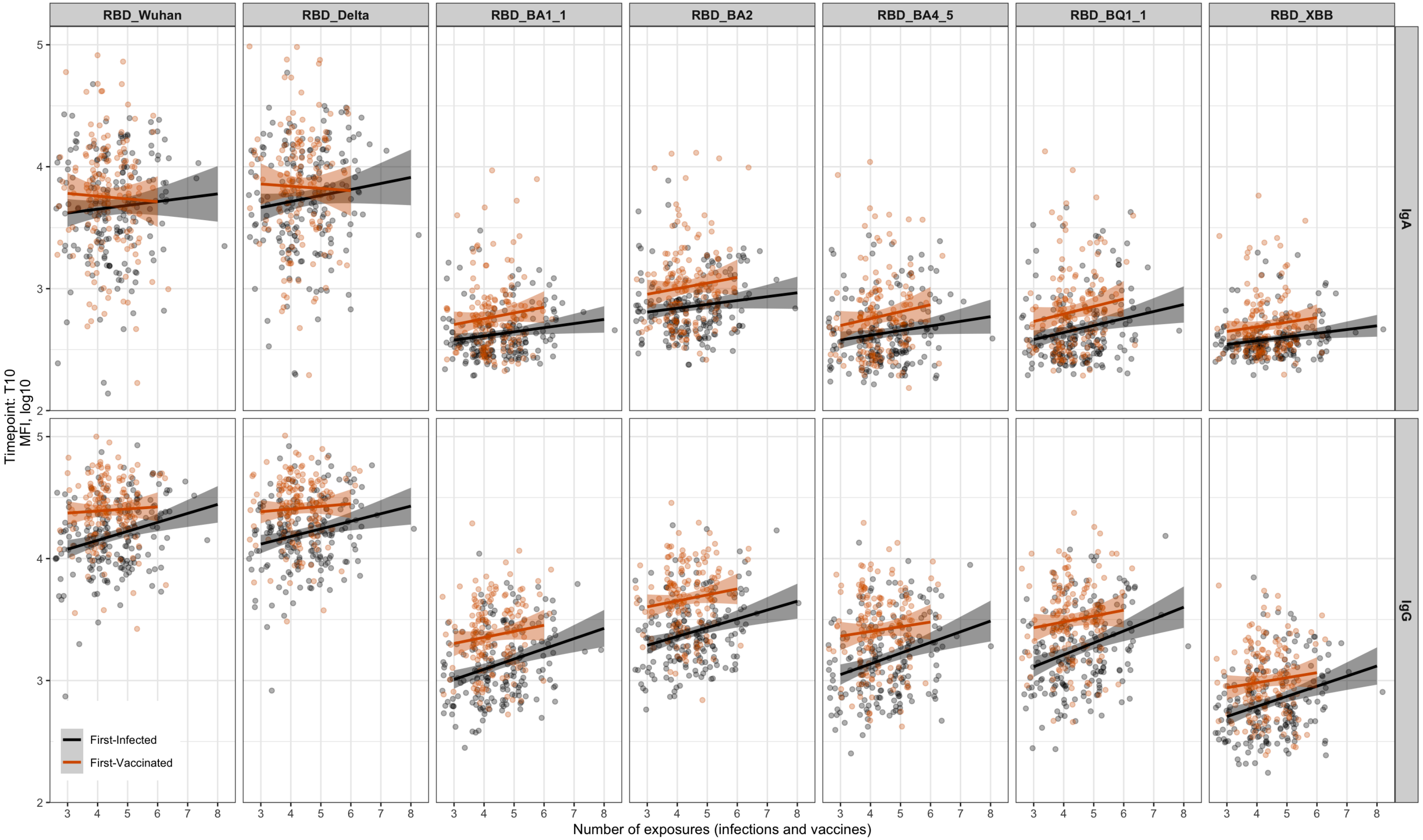

Shaded ribbons represents 95% confidence intervals.

At T11

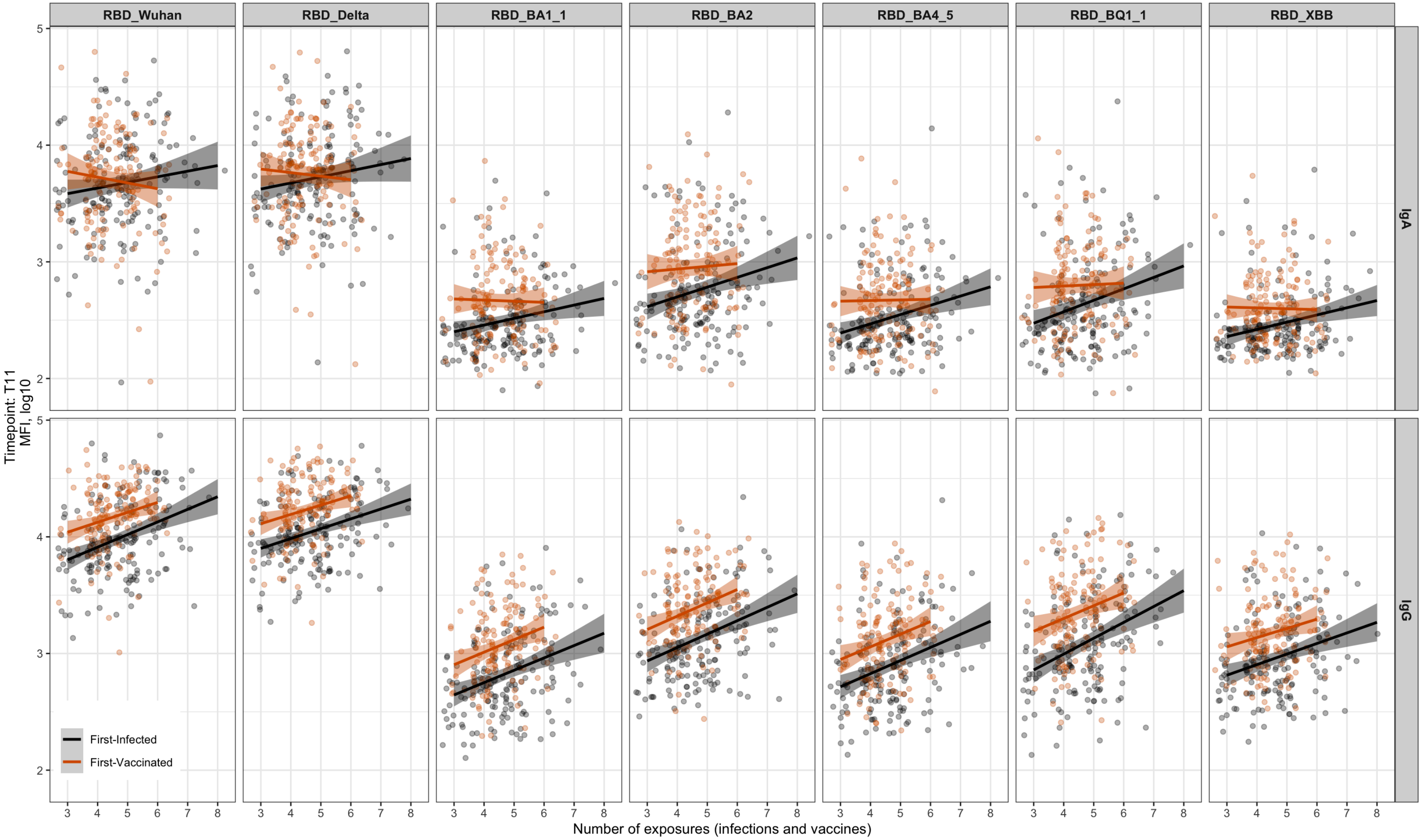

Shaded ribbons represents 95% confidence intervals.

Supplementary eTable 2. Mean difference on antibodies levels at timepoints T10 and T11 in the first-vaccinated vs the first-infected for infection and vaccine covariates from the main model (Model 5).

For IgA

Isotype	Variable domain	Covariate	RBD	RBD-Delta	RBD-BA1.1	RBD-BA.2	RBD-BA4/5	RBD-BQ1.1	RBD-XBB
IgA	Infection	One Sympt. Omicron	1 (-18 to 25)	3 (-16 to 28)	3 (-12 to 19)	5 (-12 to 25)	5 (-11 to 23)	13 (-6 to 34)	1 (-11 to 15)
		Two Sympt. Omicron	-9 (-36 to 29)	-10 (-37 to 28)	-14 (-34 to 11)	-3 (-28 to 33)	-14 (-35 to 14)	16 (-14 to 58)	-12 (-30 to 10)
		One Symp. non-Omicron	-29 (-54 to 8)	-20 (-47 to 21)	-15 (-35 to 13)	-21 (-43 to 9)	-13 (-35 to 17)	-14 (-38 to 19)	-9 (-28 to 16)
		One Asymp. Omicron	-11 (-31 to 15)	-8 (-29 to 19)	4 (-12 to 23)	8 (-12 to 31)	6 (-11 to 28)	17 (-5 to 43)	5 (-9 to 22)
		Two Asymp. Omicron	59 (-60 to 525)	61 (-58 to 522)	46 (-40 to 255)	89 (-33 to 436)	46 (-44 to 278)	109 (-27 to 497)	44 (-32 to 207)
		One Asymp. non-Omicron	-6 (-29 to 26)	-4 (-28 to 28)	9 (-10 to 32)	8 (-13 to 35)	4 (-15 to 27)	15 (-8 to 44)	6 (-10 to 24)
		Two Asymp. non-Omicron	47 (-62 to 479)	56 (-60 to 499)	-3 (-60 to 137)	24 (-56 to 251)	-21 (-70 to 104)	-28 (-75 to 106)	-19 (-62 to 72)
	Vaccine	Two non-Bivalent doses	20 (-12 to 64)	23 (-10 to 68)	**28 (4 to 57)**	**37 (8 to 75)**	**34 (8 to 67)**	23 (-3 to 57)	**20 (0 to 42)**
		Three non-Bivalent doses	18 (-17 to 68)	23 (-13 to 74)	22 (-4 to 53)	24 (-5 to 63)	26 (-2 to 62)	23 (-7 to 61)	**23 (1 to 50)**
		Four non-Bivalent doses	82 (-13 to 278)	69 (-20 to 259)	34 (-23 to 136)	86 (-5 to 265)	69 (-9 to 215)	**95 (0 to 279)**	40 (-16 to 134)
		One Bivalent dose	**27 (5 to 53)**	**26 (4 to 52)**	9 (-5 to 26)	**27 (8 to 51)**	**25 (7 to 46)**	**37 (16 to 62)**	6 (-6 to 21)

Estimates from linear mixed models estimating the %MFI increase. The model accounted for repeated measurements in the same individual with a random intercept per individual. The model was adjusted as the main model (M5) and included, first exposure, age (restricted cubic spline with 3df) + sex + number of chronic comorbidities and tobacco smoking status + number of non-Omicron and Omicron symptomatic infections (as factors) + number of non-Omicron and Omicron asymptomatic infections (as factors) + number of non-bivalent and bivalent vaccines (as factors) + days from last infection (restricted cubic spline with 3df) + days from last vaccine (restricted cubic spline with 3df).

For IgG

Isotype	Variable domain	Covariate	RBD	RBD-Delta	RBD-BA1.1	RBD-BA.2	RBD-BA4/5	RBD-BQ1.1	RBD-XBB
IgG	Infection	One Sympt. Omicron	-4 (-17 to 12)	-4 (-17 to 11)	13 (-4 to 34)	5 (-11 to 23)	9 (-8 to 30)	16 (-3 to 38)	**28 (9 to 49)**
		Two Sympt. Omicron	-12 (-33 to 16)	-9 (-30 to 18)	11 (-19 to 51)	7 (-21 to 45)	6 (-24 to 47)	37 (-1 to 90)	**85 (40 to 146)**
		One Symp. non-Omicron	-14 (-34 to 12)	-10 (-30 to 16)	-22 (-41 to 3)	-29 (-46 to -7)	-19 (-40 to 9)	-21 (-42 to 8)	-15 (-36 to 12)
		One Asymp. Omicron	2 (-14 to 19)	5 (-10 to 23)	**22 (2 to 44)**	11 (-6 to 31)	**24 (3 to 48)**	**31 (8 to 58)**	**36 (15 to 61)**
		Two Asymp. Omicron	22 (-47 to 180)	13 (-49 to 152)	3 (-57 to 147)	39 (-41 to 226)	3 (-60 to 164)	26 (-53 to 236)	10 (-55 to 165)
		One Asymp. non-Omicron	7 (-11 to 28)	5 (-12 to 25)	6 (-13 to 28)	4 (-13 to 26)	1 (-18 to 24)	5 (-15 to 30)	-4 (-20 to 17)
		Two Asymp. non-Omicron	48 (-36 to 238)	44 (-35 to 220)	-8 (-61 to 121)	2 (-57 to 140)	-22 (-69 to 101)	-26 (-72 to 97)	-18 (-66 to 98)
	Vaccine	Two non-Bivalent doses	**33 (9 to 61)**	**28 (6 to 54)**	**35 (10 to 66)**	**28 (5 to 57)**	**29 (4 to 61)**	**27 (1 to 59)**	**24 (1 to 52)**
		Three non-Bivalent doses	**64 (32 to 105)**	**38 (12 to 70)**	**55 (23 to 96)**	**53 (22 to 92)**	**48 (15 to 90)**	**55 (19 to 100)**	**44 (14 to 82)**
		Four non-Bivalent doses	**98 (5 to 274)**	46 (-19 to 164)	**116 (3 to 356)**	**108 (2 to 323)**	95 (-10 to 324)	**120 (5 to 363)**	**179 (48 to 427)**
		One Bivalent dose	**20 (3 to 40)**	**30 (12 to 50)**	19 (-1 to 42)	**28 (8 to 51)**	**32 (10 to 59)**	**43 (20 to 72)**	**64 (40 to 91)**

Estimates from linear mixed models estimating the %MFI increase. The model accounted for repeated measurements in the same individual with a random intercept per individual. The model was adjusted as the main model (M5) and included, first exposure, age (restricted cubic spline with 3df) + sex + number of chronic comorbidities and tobacco smoking status + number of non-Omicron and Omicron symptomatic infections (as factors) + number of non-Omicron and Omicron asymptomatic infections (as factors) + number of non-bivalent and bivalent vaccines (as factors) + days from last infection (restricted cubic spline with 3df) + days from last vaccine (restricted cubic spline with 3df).

Supplementary eFigure 3. Percent change on IgA and IgG anti-RBD/anti-S ratio at timepoints T10 and T11 in first-vaccinated group minus first-infected group*. RBD: Receptor binding domain. S: Spike.

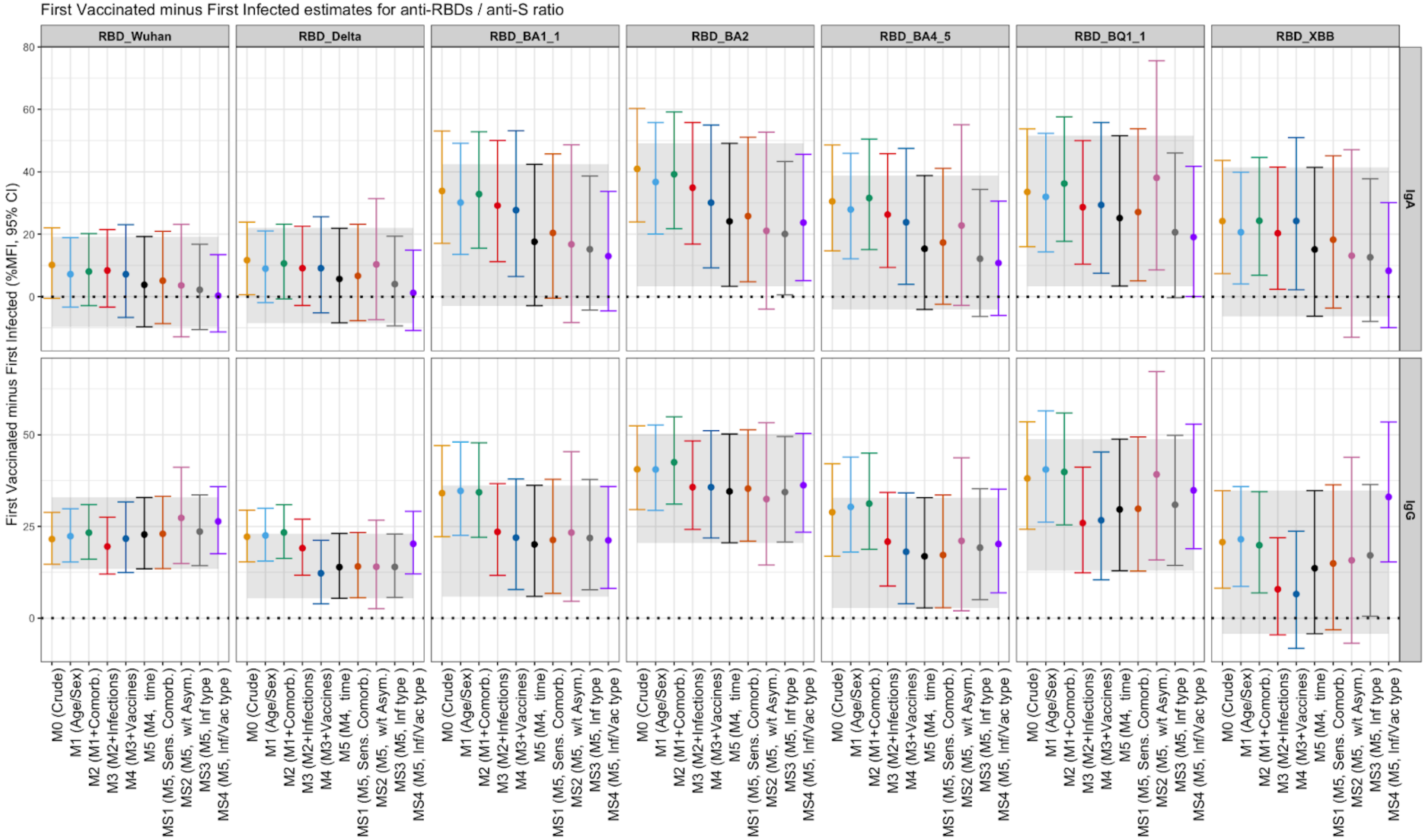

* Estimates from linear mixed models estimating the percent change increase in those first-vaccinated compared to the first-infected group (reference). The models accounted for repeated measurements in the same individual with a random intercept per individual. The models were adjusted as follows: M0, crude; M1, M0 + age (restricted cubic spline with 3df) and sex; M2, M1 + number of chronic comorbidities and tobacco smoking status; M3, M2 + number of non-Omicron and Omicron symptomatic infections (as factors), and number of non-Omicron and Omicron asymptomatic infections (as factors); M4, M3 + number of non-bivalent and bivalent vaccines (as factors); and finally, M5 (main model), M4 + days from last infection (restricted cubic spline with 3df) and days from last vaccine (restricted cubic spline with 3df). MS1, M5 but expanding the number of chronic comorbidities as three binary factors (cardio-metabolic, immunosuppressed and previous allergy); MS2, running M5 in those individuals without any history of asymptomatic infections; MS3, M5 but instead of non-bivalent/valent, using number of mRNA and adenovirus-based vaccines; MS4, MS3 but instead of number of non-Omicron/Omicron, using number of previous infections by each variant.

Supplementary eFigure 4. Comparison of different methods of p-value correction for multiplicity in the analysis of full sequence history of previous infections and vaccines.

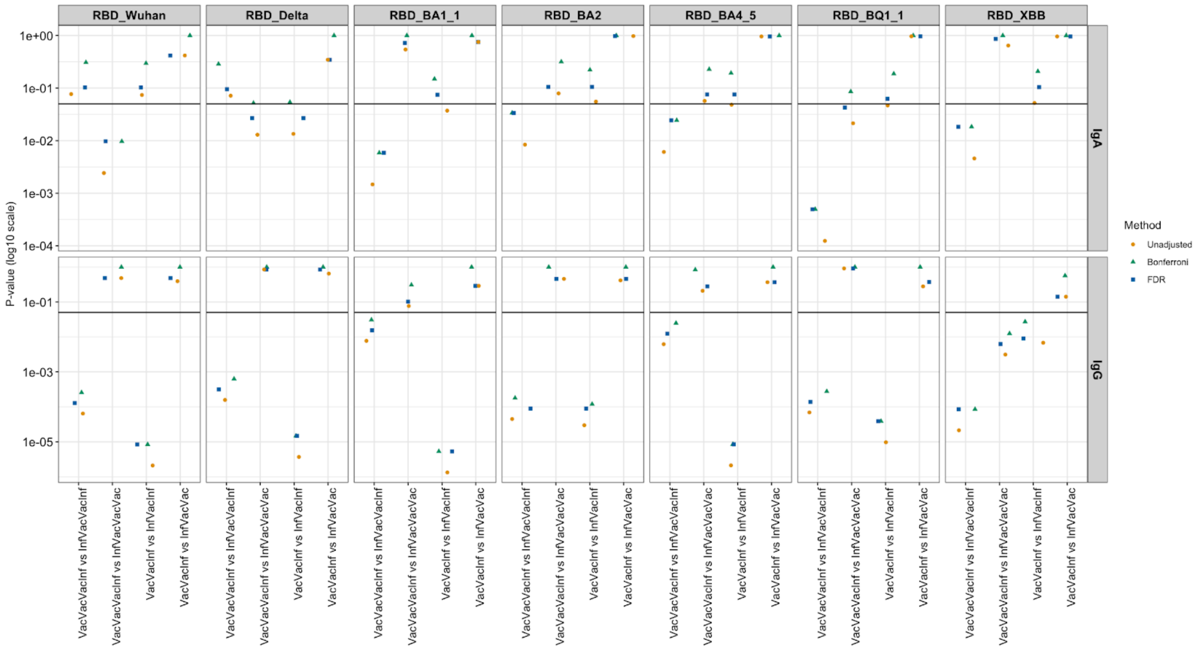

The horizontal black lines represent the threshold of p-value <0.05. FDR denotes false discovery rate.

P-values for each exposure history contrast and different methods of adjust for multiple comparisons.

Contrast	Variant	IgA			IgG		
		Unadjusted	Bonferroni	FDR	Unadjusted	Bonferroni	FDR
VacVacInf vs InfVacVac	RBD_Wuhan	0.4165	>0.9999	0.4165	0.3921	>0.9999	0.482
VacVacInf vs InfVacInf	RBD_Wuhan	0.0741	0.2963	0.1028	<0.0001	<0.0001	<0.0001
VacVacVacInf vs InfVacVacVac	RBD_Wuhan	0.0024	0.0097	0.0097	0.482	>0.9999	0.482
VacVacVacInf vs InfVacVacInf	RBD_Wuhan	0.0771	0.3084	0.1028	0.0001	0.0003	0.0001
VacVacInf vs InfVacVac	RBD_Delta	0.3463	>0.9999	0.3463	0.6481	>0.9999	0.8518
VacVacInf vs InfVacInf	RBD_Delta	0.0135	0.0539	0.027	<0.0001	<0.0001	<0.0001
VacVacVacInf vs InfVacVacVac	RBD_Delta	0.013	0.052	0.027	0.8518	>0.9999	0.8518
VacVacVacInf vs InfVacVacInf	RBD_Delta	0.0716	0.2865	0.0955	0.0002	0.0006	0.0003
VacVacInf vs InfVacVac	RBD_BA1_1	0.7526	>0.9999	0.7526	0.2899	>0.9999	0.2899
VacVacInf vs InfVacInf	RBD_BA1_1	0.0373	0.1492	0.0746	<0.0001	<0.0001	<0.0001
VacVacVacInf vs InfVacVacVac	RBD_BA1_1	0.541	>0.9999	0.7213	0.0768	0.3071	0.1024
VacVacVacInf vs InfVacVacInf	RBD_BA1_1	0.0015	0.0059	0.0059	0.0077	0.0309	0.0155
VacVacInf vs InfVacVac	RBD_BA2	0.9754	>0.9999	0.9754	0.4153	>0.9999	0.4577
VacVacInf vs InfVacInf	RBD_BA2	0.0555	0.2218	0.1057	<0.0001	0.0001	0.0001
VacVacVacInf vs InfVacVacVac	RBD_BA2	0.0793	0.3171	0.1057	0.4577	>0.9999	0.4577
VacVacVacInf vs InfVacVacInf	RBD_BA2	0.0084	0.0336	0.0336	<0.0001	0.0002	0.0001
VacVacInf vs InfVacVac	RBD_BA4_5	0.9578	>0.9999	0.9578	0.3677	>0.9999	0.3677
VacVacInf vs InfVacInf	RBD_BA4_5	0.0483	0.1933	0.076	<0.0001	<0.0001	<0.0001
VacVacVacInf vs InfVacVacVac	RBD_BA4_5	0.057	0.2279	0.076	0.2092	0.8366	0.2789
VacVacVacInf vs InfVacVacInf	RBD_BA4_5	0.0061	0.0244	0.0244	0.0062	0.0249	0.0124
VacVacInf vs InfVacVac	RBD_BQ1_1	0.9648	>0.9999	0.9648	0.2791	>0.9999	0.3721
VacVacInf vs InfVacInf	RBD_BQ1_1	0.0469	0.1874	0.0625	<0.0001	<0.0001	<0.0001
VacVacVacInf vs InfVacVacVac	RBD_BQ1_1	0.0215	0.0858	0.0429	0.9103	>0.9999	0.9103
VacVacVacInf vs InfVacVacInf	RBD_BQ1_1	0.0001	0.0005	0.0005	0.0001	0.0003	0.0001
VacVacInf vs InfVacVac	RBD_XBB	0.9578	>0.9999	0.9578	0.1416	0.5662	0.1416
VacVacInf vs InfVacInf	RBD_XBB	0.0521	0.2085	0.1042	0.0068	0.0273	0.0091
VacVacVacInf vs InfVacVacVac	RBD_XBB	0.6451	>0.9999	0.8602	0.0031	0.0126	0.0063
VacVacVacInf vs InfVacVacInf	RBD_XBB	0.0046	0.0184	0.0184	<0.0001	0.0001	0.0001

The three situations where the multiplicity adjustment did a significant change in the interpretation are highlighted in yellow.
The p-value tests were all two-sided.

Supplementary eFigure 5. Longitudinal Wuhan antibody levels against nucleocapsid (N), receptor-binding domain (RBD), and spike (S) proteins overtime applying different parametrizations and models (sensitivity analyses)

Panel A. Robustness of antibody trajectories to smoothing procedure. a. Robust LOESS (span = 0.5, degree = 1 (local linear fit), symmetric (robust to outliers)). **b.** Span sensitivity for LOESS (degree = 2, gaussian) (span: 0.4–0.7). **c.** Span sensitivity for LOESS (degree = 1, symmetric) (span: 0.4–0.7).

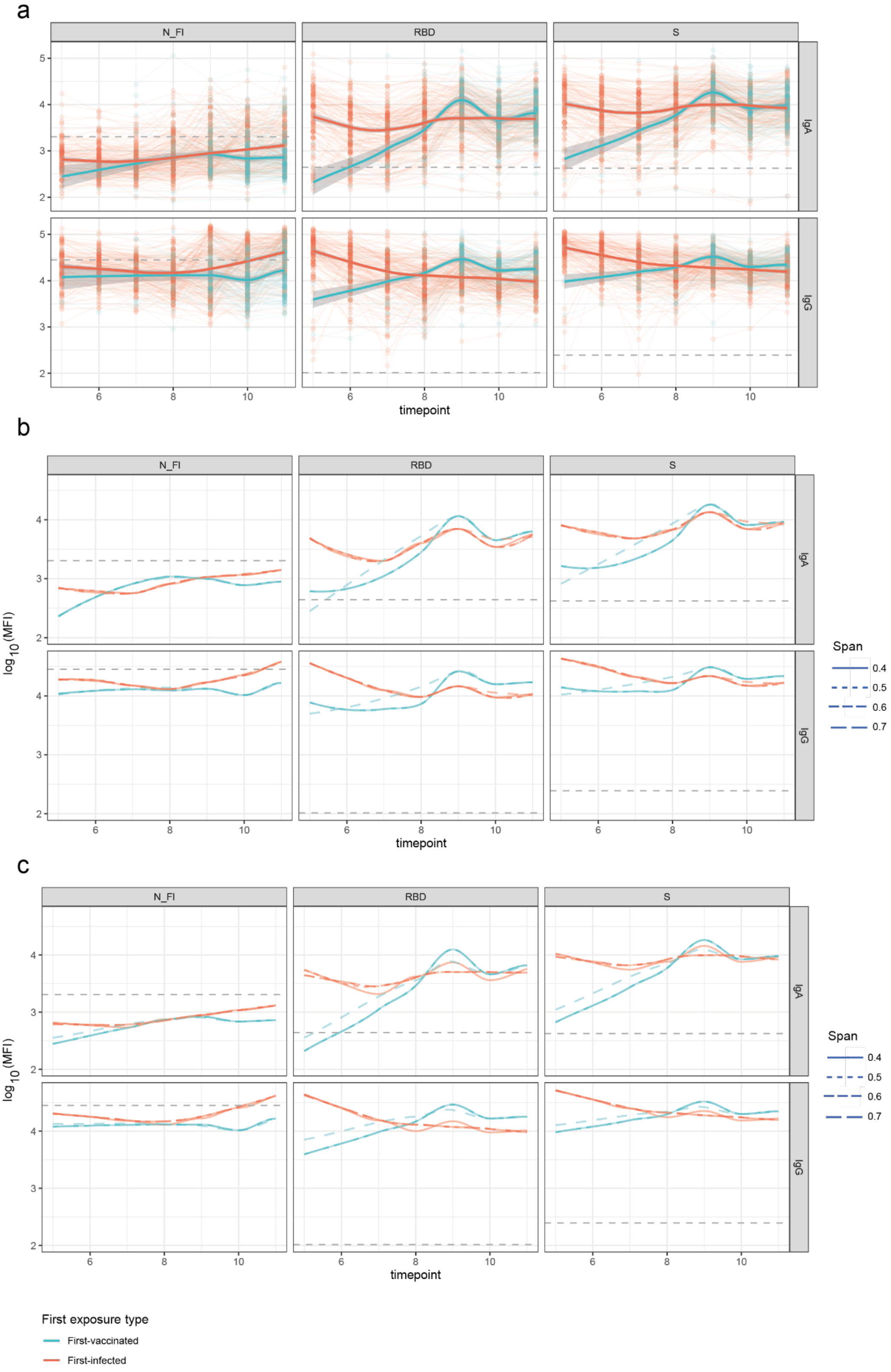

Panel B. Robustness of antibody trajectories to smoothing procedure. a. Participant-level cluster bootstrap (300 iterations) confidence intervals (95% pointwise percentiles). b. Boundary stress evaluation (re-fitting after dropping the first or last timepoint). c. Raw data summaries: median and interquartile range of log10(MFI). MFI: Median fluorescence intensity.

a

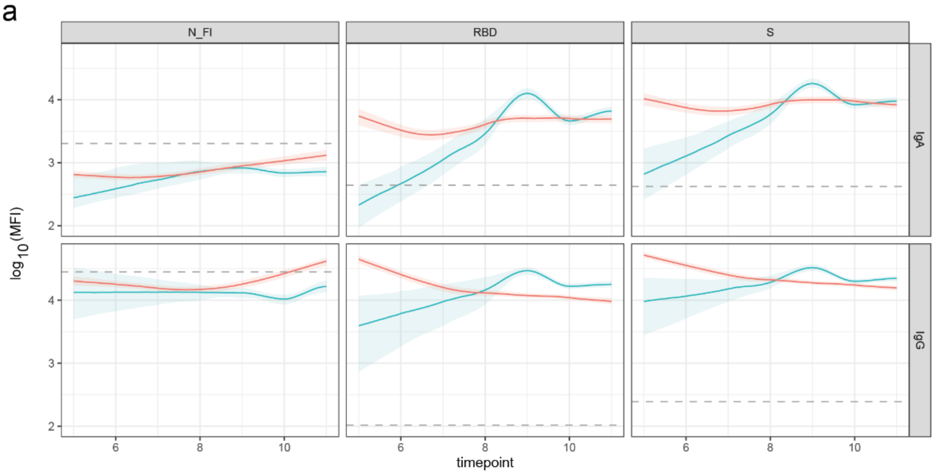

b

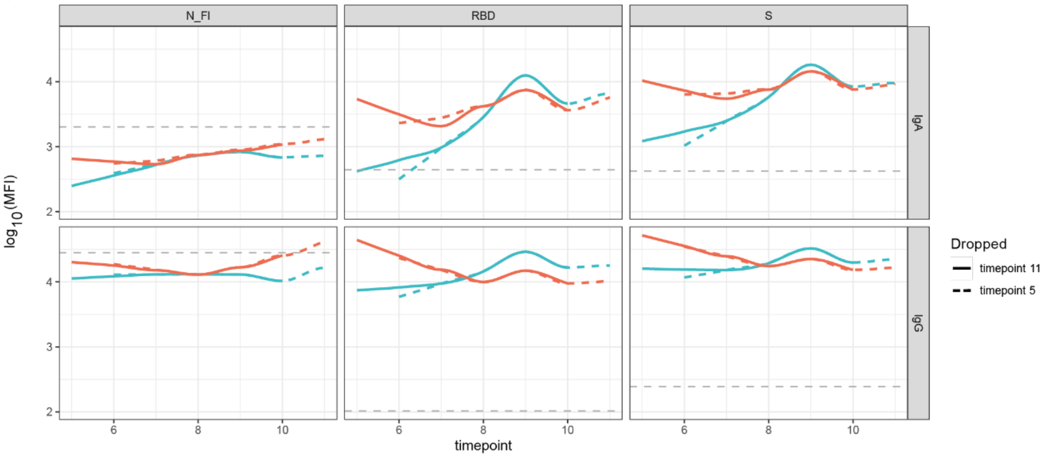

c

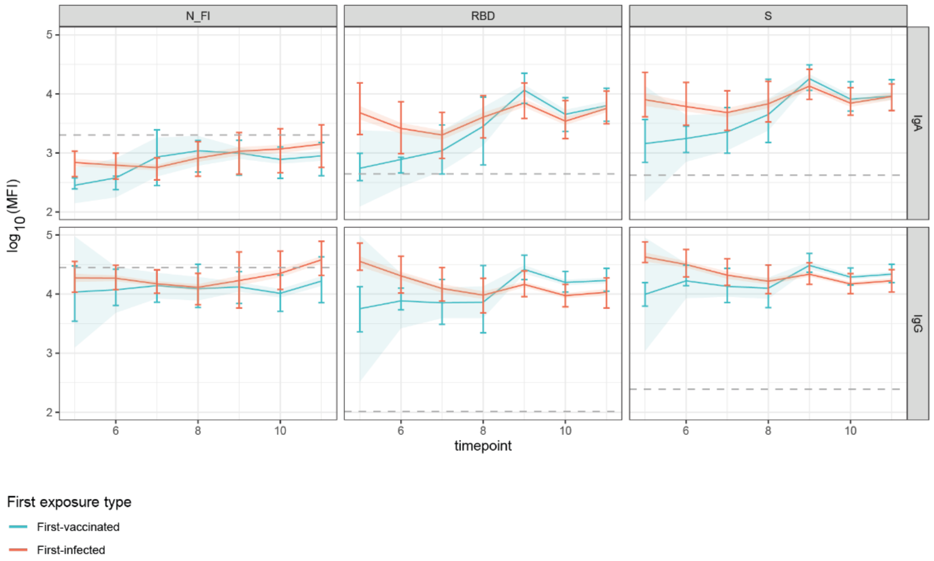

Panel C. Modelling with GAM

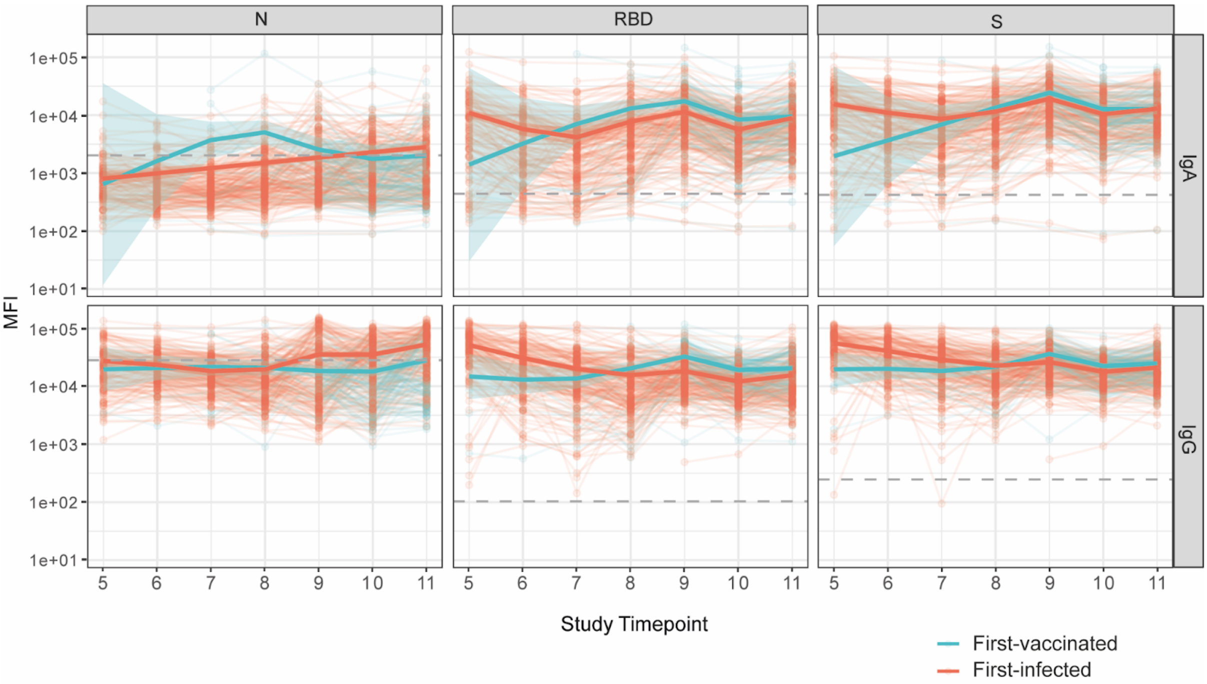

The blue and red solid lines represent the fitted curve calculated using a generalized additive model (GAM), with thin-plate penalized splines and the spline parameters were determined by the algorithm available on the R package *mgcv*. The dashed gray lines represent the mean cutoff values for each antibody and isotype. We used the mean cutoff across the timepoints to provide a single line in the figure. Cutoff values: For IgA, the mean cutoff values were 2018.49 (SD = 530.17) for the N, 441.30 (SD = 136.65) for RBD, and 421.53 (SD = 60.68) for S. For IgG, the mean cutoff values were 28128.12 (SD = 9968.65) for N, 103.33 (SD = 28.58) for RBD, and 246.40 (SD = 99.78) for S.

Supplementary eFigure 6. Magnitude of T-cell responses at T11 to S and N+M Wuhan antigens by first exposure groups. T: Timepoint.

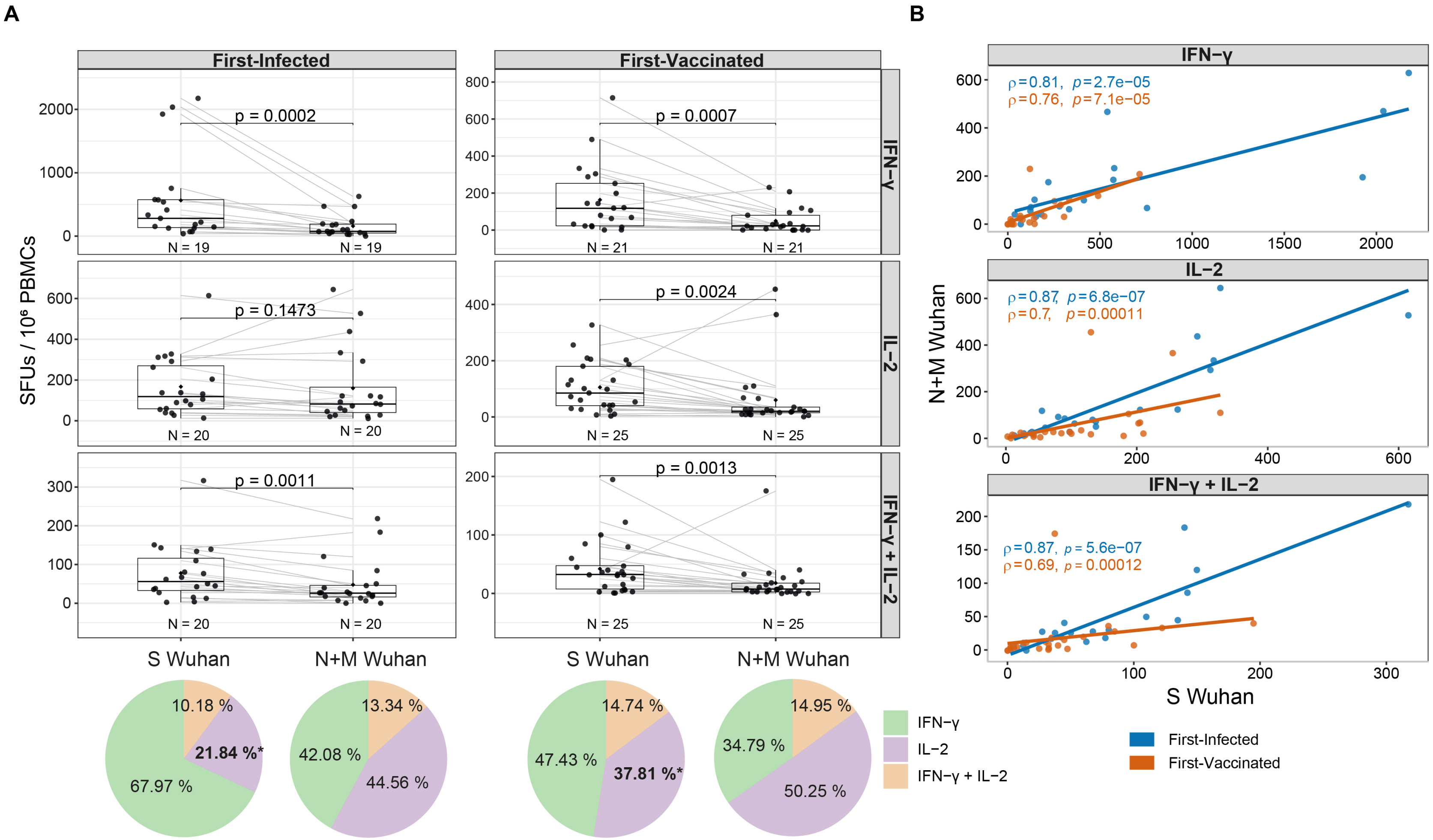

(A) Boxplots representing T-cell responses as SFUs / 10^6 peripheral blood mononuclear cells (PBMCs) secreting IFN-γ, IL-2 or IFN-γ + IL-2 (polyfunctional) and pie charts showing the average proportion (%) of secreting T cells by cytokine: IFN-γ (green), IL-2 (purple), or both (orange) to S or N+M from Wuhan by first exposure groups. Responses were compared by paired Wilcoxon Signed-Rank test. Boxplots represent median (bold line), the mean (black diamond), 1st and 3rd quartiles (box), and largest and smallest values within 1.5 times the interquartile range (whiskers). Proportions were compared by Chi-square test, statistically significant differences p-values: * ≤ 0.05 . (B) Spearman's correlation coefficient ρ (Rho) between the magnitude of T-cell responses to S and N+M antigens. Seven participants with ≥100 SFU in unstimulated wells for IFN-γ were excluded from the analysis. IFN-γ: Interferon-gamma (IFN-γ),. IL-2: Iinterleukin-2. (IL-2), M: Mmembrane (M),. N: nNucleocapsid (N),. RBD: rReceptor binding domain (RBD), S: Sspike (S),. SFU: Sspot-forming units (SFU).

Supplementary eTable3. Unadjusted and adjusted Cox Proportional Hazard model for the association between first exposure (first-vaccinated/first-infected) and breakthrough infections

	Number of events/Number at Risk	Person-years	HR (95% CI) Unadjusted	HR (95% CI) Adjusted*
**Main analysis**				
Period T8-T9	29/182	31.4	1.63 (0.66 - 4.01)	3.48 (1.05 - 11.5)
Period T9-T10	83/263	105.4	0.66 (0.41 - 1.08)	0.50 (0.31 - 0.82)
Period T10-T11	68/280	106.2	0.81 (0.50 - 1.33)	0.71 (0.42 - 1.17)
**Sensitivity analysis, without considering asymptomatic infections**				
Period T8-T9	25/178	30.4	1.89 (0.73 - 4.63)	4.97 (1.41 - 17.5)
Period T9-T10	55/235	98.1	0.53 (0.27 - 1.01)	0.46 (0.24 - 0.88)
Period T10-T11	28/240	96.6	0.54 (0.23 - 1.22)	0.44 (0.19 - 1.02)

* Model adjusted for age, sex, number of comorbidities, smoking status, number of previous exposures, and time since last exposure.

Supplementary eFigure 7. Correlation between anti-RBD and neutralizing antibodies measured in a subset of individuals (n=89) from the cohort at T11. RBD: receptor binding domain. T: timepoint.

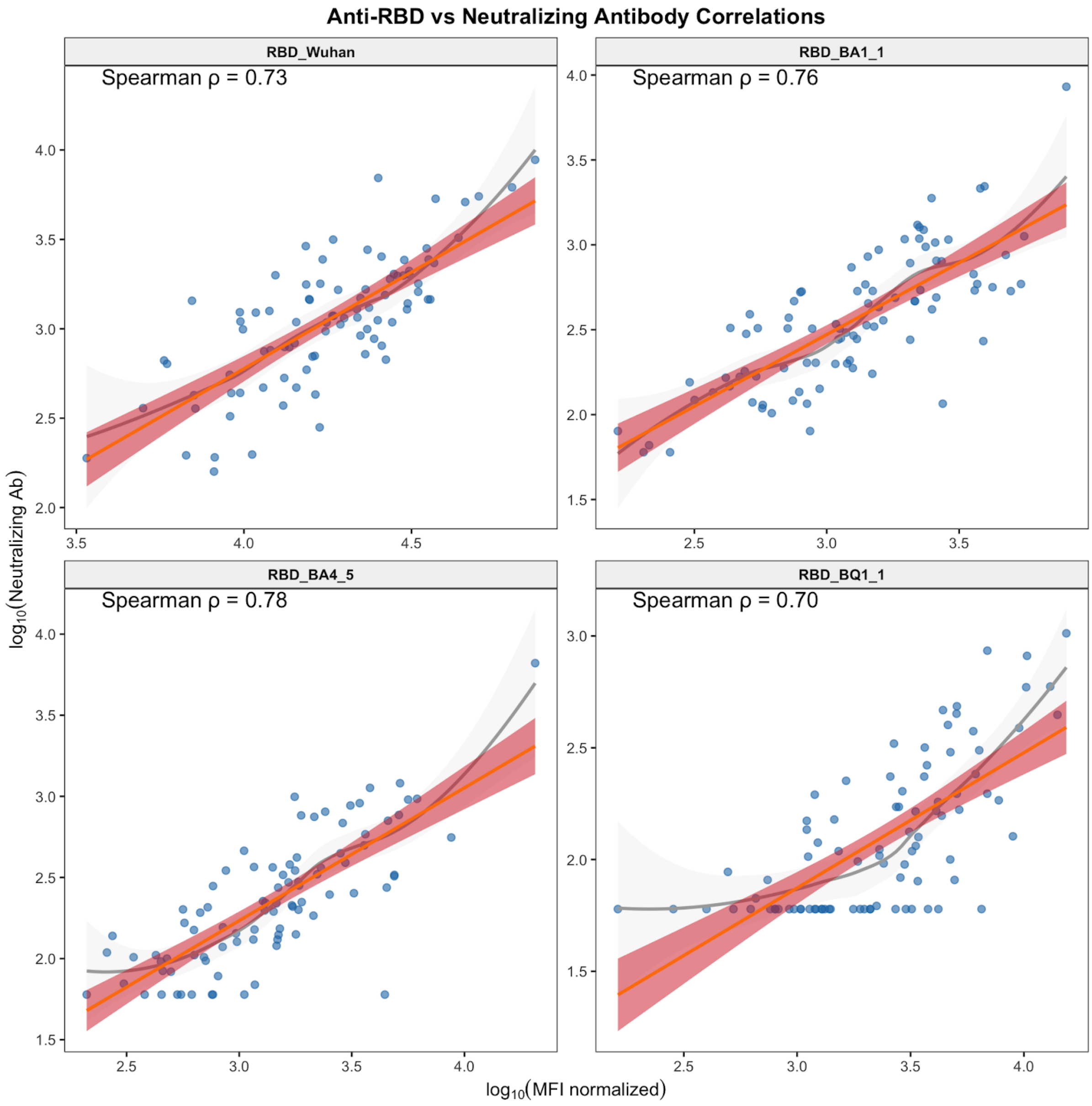

The red lines represent the linear fit and the dark-grey lines represent the LOWESS fit.

Supplementary eTable 4. Summary of linear mixed models used to estimate the association between first exposure (i.e., First-vaccinated *minus* First-infected) and anti-RBDs. RBD: Receptor binding domain.

Model	Model	Adjustment
M0	Crude	None
M1	Adjust by age and sex	Age + Sex
M2	M1 + adjust by comorbidities and behaviour	Age + Sex + Number of chronic comorbidities + tobacco smoking status
M3	M2 + adjust by previous infections history	Age + Sex + Number of chronic comorbidities + tobacco smoking status + number of previous non-Omicron symptomatic infections + number of previous Omicron symptomatic infections + number of previous non-Omicron asymptomatic infections + number of previous Omicron asymptomatic infections
M4	M3 + adjust by previous vaccines history	Age + Sex + Number of chronic comorbidities + tobacco smoking status + number of previous non-Omicron symptomatic infections + number of previous Omicron symptomatic infections + number of previous non-Omicron asymptomatic infections + number of previous Omicron asymptomatic infections + number of previous non-bivalent vaccines + number of previous bivalent vaccines
M5 (main model)	M4 + adjust by time from the last previous exposure	Age + Sex + Number of chronic comorbidities + tobacco smoking status + number of previous non-Omicron symptomatic infections + number of previous Omicron symptomatic infections + number of previous non-Omicron asymptomatic infections + number of previous Omicron asymptomatic infections + number of previous non-bivalent vaccines + number of previous bivalent vaccines + days from last infection + days from last vaccine
MS1	M5, but using specific comorbidities instead of number of comorbidities	Age + Sex + cardio-metabolic comorbidity + immunosuppression status + allergy + tobacco smoking status + number of previous non-Omicron symptomatic infections + number of previous Omicron symptomatic infections + number of previous non-Omicron asymptomatic infections + number of previous Omicron asymptomatic infections + number of previous non-bivalent vaccines + number of previous bivalent vaccines + days from last infection + days from last vaccine
MS2	M5 among those without any previous asymptomatic infection	Age + Sex + Number of chronic comorbidities + tobacco smoking status + number of previous non-Omicron symptomatic infections + number of previous Omicron symptomatic infections + number of previous non-bivalent vaccines + number of previous bivalent vaccines + days from last infection + days from last vaccine
MS3	M5, but using VoC as previous infection	Age + Sex + Number of chronic comorbidities + tobacco smoking status + number of previous Wuhan + number of previous Alpha + number of previous Delta + number of previous BA1 + number of previous BA2 + number of previous BA.4/5+ number of previous BQ.1.1 + number of previous XBB + number of previous non-bivalent vaccines + number of previous bivalent vaccines + days from last infection + days from last vaccine
MS4	MS3, but using type of previous vaccine	Age + Sex + Number of chronic comorbidities + tobacco smoking status + number of previous Wuhan + number of previous Alpha + number of previous Delta + number of previous BA1 + number of previous BA2 + number of previous BA.4/5+ number of previous BQ.1.1 + number of previous XBB + number of previous mRNA vaccines + number of previous adenovirus-based vaccines + days from last infection + days from last vaccine

Supplementary eFigure 8. Variance Inflation Factors (VIFs) from the main model (M5) for IgA and IgG and the seven RBDs. RBD: Receptor binding domain.

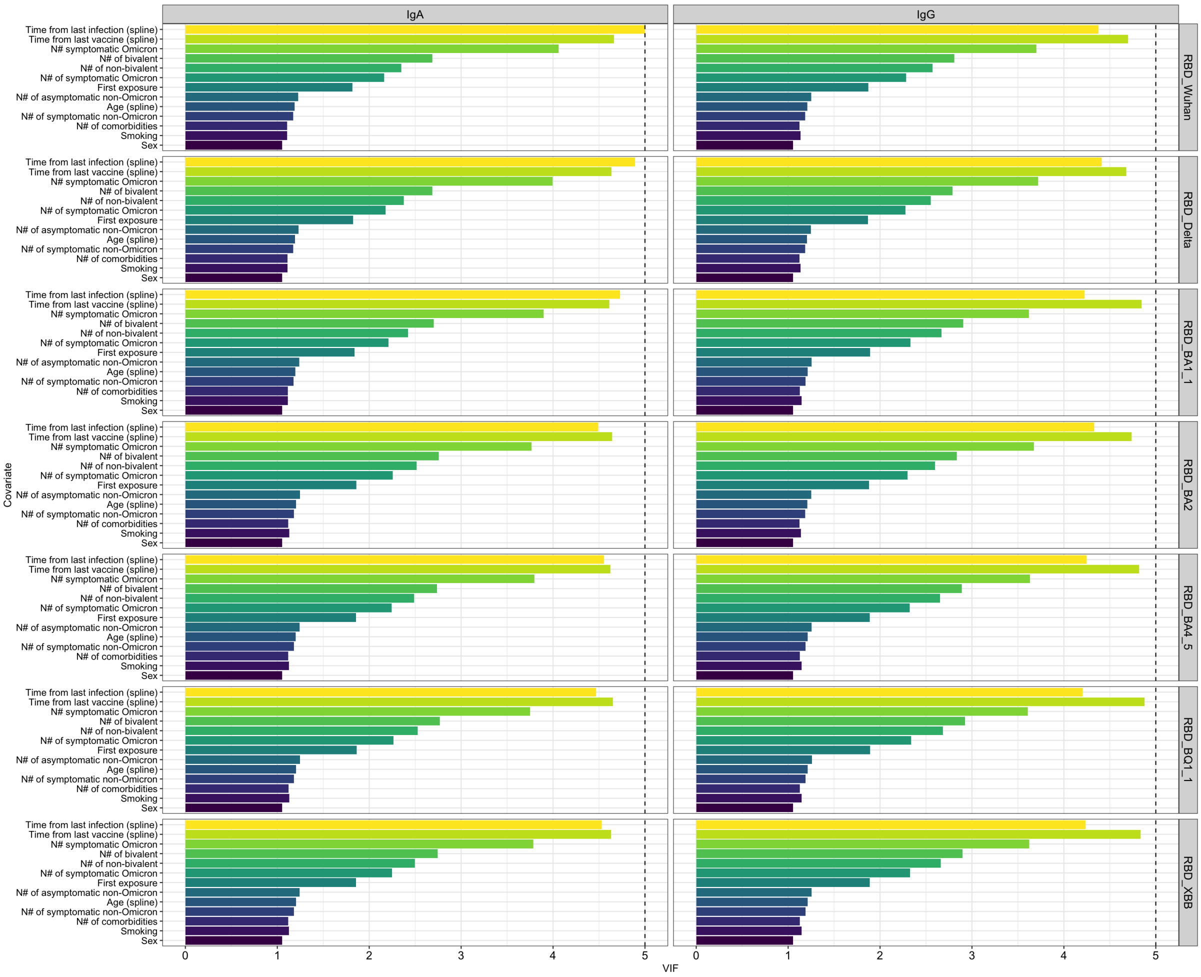

The vertical dashed black lines are the VIF of 5, which might indicate multicollinearity. We plotted the spline terms for age, time from last infection and time from last vaccine, nevertheless the interpretation of VIF values for spline terms is not straightforward.

Supplementary eTable 5. Parametrizations for the antibody kinetics for IgA and IgG against Wuhan with anti-S, anti-N and anti-RBD antibodies using the data available from T5 to T11. S: Spike. N: Nucleocapsid. RBD: Receptor binding domain. T: Timepoint.

**Method**	**Parameters**	**Comment**
locally weighted scatterplot smoothing (LOWESS)	degree: "2 - quadratic"; family: "gaussian"; surface: "interpolate", span 0.50	Main analysis
locally weighted scatterplot smoothing (LOWESS)	degree: "1 - linear"; family: "symmetric"; surface: "interpolate", span 0.50	Aimed to reduce sensitivity to local outliers and down-weight boundary influence
locally weighted scatterplot smoothing (LOWESS)	degree: "2 - quadratic"; family: "gaussian"; surface: "interpolate", span between 0.4-0.7	Span sensitivity
locally weighted scatterplot smoothing (LOWESS)	degree: "2 - quadratic"; family: "gaussian"; surface: "interpolate", span 0.50, with participant-level bootstrap, resampling individuals with replacement, refitting the smoother on each resample, and constructing percentile-based confidence bands	Cluster bootstrap by participant
locally weighted scatterplot smoothing (LOWESS)	degree: "2 - quadratic"; family: "gaussian"; surface: "interpolate", span 0.50 without the first (T5) and last (T11) observation	Boundary stress test
Generalized Additive Model (GAM)	thin-plate splines and smoothing parameters selected by the *mgcv* algorithm	
