# Supplementary material for: Primary SARS-CoV-2 exposure by vaccination or infection shapes immune responses to omicron variants among a Spanish cohort
Source: Nat Commun. 2025 Dec 13;17:863. doi: 10.1038/s41467-025-67577-9 (PMC12827448; doi:10.1038/s41467-025-67577-9)
Supplement: Supplementary file 2 — Reporting Summary [file 41467_2025_67577_MOESM2_ESM.pdf]

nature portfolio

Corresponding author(s): Otavio Ranzani / Carlota Dobaño

Last updated by author(s): Nov 21, 2025

Reporting Summary

Nature Portfolio wishes to improve the reproducibility of the work that we publish. This form provides structure for consistency and transparency in reporting. For further information on Nature Portfolio policies, see our Editorial Policies and the Editorial Policy Checklist.

Statistics

For all statistical analyses, confirm that the following items are present in the figure legend, table legend, main text, or Methods section.

n/a	Confirmed	
☐	☒	The exact sample size (*n*) for each experimental group/condition, given as a discrete number and unit of measurement
☐	☒	A statement on whether measurements were taken from distinct samples or whether the same sample was measured repeatedly
☐	☒	The statistical test(s) used AND whether they are one- or two-sided<br>*Only common tests should be described solely by name; describe more complex techniques in the Methods section.*
☐	☒	A description of all covariates tested
☐	☒	A description of any assumptions or corrections, such as tests of normality and adjustment for multiple comparisons
☐	☒	A full description of the statistical parameters including central tendency (e.g. means) or other basic estimates (e.g. regression coefficient) AND variation (e.g. standard deviation) or associated estimates of uncertainty (e.g. confidence intervals)
☐	☒	For null hypothesis testing, the test statistic (e.g. *F*, *t*, *r*) with confidence intervals, effect sizes, degrees of freedom and *P* value noted<br>*Give P values as exact values whenever suitable.*
☒	☐	For Bayesian analysis, information on the choice of priors and Markov chain Monte Carlo settings
☒	☐	For hierarchical and complex designs, identification of the appropriate level for tests and full reporting of outcomes
☐	☒	Estimates of effect sizes (e.g. Cohen's *d*, Pearson's *r*), indicating how they were calculated

Our web collection on statistics for biologists contains articles on many of the points above.

Software and code

Policy information about availability of computer code

Data collection	Demographic and clinical data for each participant were collected at baseline and during follow-up visits through telephone interviews and electronic standardized questionnaires by study physicians and nurses in RedCap version 8.8.2)
Data analysis	We performed the statistical analysis in the software R version 4.2.1. No custom code or algorithms were developed for this study.

For manuscripts utilizing custom algorithms or software that are central to the research but not yet described in published literature, software must be made available to editors and reviewers. We strongly encourage code deposition in a community repository (e.g. GitHub). See the Nature Portfolio guidelines for submitting code & software for further information.

Data

Policy information about availability of data

All manuscripts must include a data availability statement. This statement should provide the following information, where applicable:

- Accession codes, unique identifiers, or web links for publicly available datasets
- A description of any restrictions on data availability
- For clinical datasets or third party data, please ensure that the statement adheres to our policy

In accordance with the current European and national law, the data used in this study are only available for the researchers participating in this study. Thus, we are not allowed to distribute or make publicly available the data to other parties. Researchers wishing to obtain the data must submit a request to the corresponding data custodian (Prof. Carlota Dobaño; carlota.dobano@isglobal.org). Requests will be evaluated within one month. Access will be granted only for research purposes that are compatible with the original ethics approval and will require (i) evidence of institutional affiliation, (ii) a data-use agreement, and (iii) approval

from a relevant ethics committee if newly mandated by the proposed use. All shared data will be provided in de-identified form, and use will be restricted to the purposes agreed upon in the data-use agreement.

Research involving human participants, their data, or biological material

Policy information about studies with human participants or human data. See also policy information about sex, gender (identity/presentation), and sexual orientation and race, ethnicity and racism.

Reporting on sex and gender	We report on self-reported sex, and accounted for it in the modelling strategy.
Reporting on race, ethnicity, or other socially relevant groupings	We did not have any data on race, ethnicity, or other socially relevant groupings.
Population characteristics	They were health-care workers from the primary care sector, with an average age of 50 yo and more than 80% were females.
Recruitment	These healthcare workers were recruited from three health units in Catalonia region.
Ethics oversight	The study protocols were approved by the IRB Comitè Ètic d'Investigació Clínica IDIAP Jordi Gol (20/162-PCV). Written informed consent was obtained from all study participants prior to study initiation.

Note that full information on the approval of the study protocol must also be provided in the manuscript.

Field-specific reporting

Please select the one below that is the best fit for your research. If you are not sure, read the appropriate sections before making your selection.

[x] Life sciences [ ] Behavioural & social sciences [ ] Ecological, evolutionary & environmental sciences

For a reference copy of the document with all sections, see nature.com/documents/nr-reporting-summary-flat.pdf

Life sciences study design

All studies must disclose on these points even when the disclosure is negative.

Sample size	The sample size for this study was pragmatic based on the data availability of the CovidCatCentral cohort.
Data exclusions	From 405 participants with blood samples available at T10 and/or T11, we excluded 44 (13.3%) individuals representing 51 samples for not having hybrid immunity, constituted of 29 (57%) samples with 3 previous vaccines, 10 (20%) with 2 previous infections, 5 (10%) with 4 previous vaccines, 4 (8%) with 3 previous infections, 1 sample with 4 previous infections, 1 sample with 1 previous infection, and 1 sample with 2 previous vaccines. From the remaining 361 individuals with hybrid immunity, we excluded three additional participants who had fewer than three total exposures (all with only one infection and one vaccine) and one individual with an inconsistent exposure history between timepoints, which left 357 individuals and 646 samples (337 for T10 and 309 for T11).
Replication	We did all analysis using ad-hoc R files programmed by the researchers.
Randomization	There was no randomization since this is an observational study. We adjusted for potential confounders based on previous knowledge, literature and theoretical framework.
Blinding	The operators conducted the assays in a blinded manner.

Reporting for specific materials, systems and methods

We require information from authors about some types of materials, experimental systems and methods used in many studies. Here, indicate whether each material, system or method listed is relevant to your study. If you are not sure if a list item applies to your research, read the appropriate section before selecting a response.

Materials & experimental systems

n/a	Involved in the study	
[ ]	[x]	Antibodies
[ ]	[x]	Eukaryotic cell lines
[x]	[ ]	Palaeontology and archaeology
[x]	[ ]	Animals and other organisms
[x]	[ ]	Clinical data
[x]	[ ]	Dual use research of concern
[x]	[ ]	Plants

Methods

n/a	Involved in the study	
[x]	[ ]	ChIP-seq
[x]	[ ]	Flow cytometry
[x]	[ ]	MRI-based neuroimaging

Antibodies

Antibodies used	The secondary antibodies used in the qSAT assays were goat anti-human IgG conjugated to phycoerythrin (PE) (GTIG-001, Moss Bio) at a 1:400 dilution and goat anti-human IgA-PE (GTIA-001, Moss Bio) at a 1:200 dilution. To accurately quantify IgA responses, test samples and controls were pre-treated with anti-human IgG (Gullsorb) at a 1:10 dilution to prevent IgG interference.
Validation	The secondary antibodies were previously validated in each of the qSAT assays to ensure their specificity and sensitivity against the SARS-CoV-2 antigen panel, using positive, negative, and blank controls. Positive controls consisted of plasma samples from SARS-CoV-2-infected individuals at various dilutions; Negative controls were pre-pandemic plasma samples and blanks consisted of the assay buffer alone. These tests confirmed the robustness of the secondary antibodies and their reliability for use in our serological assays.

Eukaryotic cell lines

Policy information about cell lines and Sex and Gender in Research

Cell line source(s)	CHO from Santamaría (Freestyle™ 293 cells (Life technologies) and HEK 293-F (# R790-07)
Authentication	Not performed.
Mycoplasma contamination	Not tested.
Commonly misidentified lines (See ICLAC register)	NA.

Plants

Seed stocks	NA (error in the form)
Novel plant genotypes	NA (error in the form)
Authentication	NA (error in the form)
